# Metastatic prostate cancer with bone marrow infiltration mimicking multiple myeloma

**DOI:** 10.1002/ccr3.1308

**Published:** 2017-12-22

**Authors:** Pankaj Mathur, Daisy Alapat, Manoj Kumar, Sharmilan Thanendrarajan

**Affiliations:** ^1^ Myeloma Institute University of Arkansas for Medical Sciences 4301 W Markham St. Little Rock Arkansas 72205; ^2^ Department of Pathology University of Arkansas for Medical Sciences 4301 W Markham St. Little Rock Arkansas 72205; ^3^ Department of Radiology University of Arkansas for Medical Sciences 4301 W Markham St. Little Rock Arkansas 72205

**Keywords:** Multiple myeloma, plasma cell dyscrasia, prostate cancer, smoldering myeloma

## Abstract

Concomitant diagnosis of metastatic prostate cancer and a multiple myeloma in older male patients is challenging as both malignancies are usually associated with bone lesions. Exact knowledge, experience, and an interdisciplinary approach are required in order to differentiate between both malignancies and determine the exact treatment strategy.

An 85‐year‐old cachectic, male patient was referred to our institute with a 4 months history of general weakness, B‐symptoms (weight loss, night sweats), and bone pain in the thoracic and lumbar spine. The physical examination did not reveal pathological findings except cachexia. In the laboratory workup, the patient was found to have following results: hemoglobin: 13.7 g/dL (13.0–17.0 g/dL), WBC: 9.12 K/uL (3.6–9.5 K/uL), platelet: 290 K/uL (150–450 K/uL), creatinine: 1.2 mg/dL (0.6–1.3 mg/dL), sodium: 139 mmol/L (135–140 mmol/L), potassium: 4.9 mmol/L (3.5–5.1 mmol/L), calcium 9.7 mg/dL (8.6–10.2 mg/dL), AST: 35 IU/L (15–41 IU/L), and GGT: 13 IU/L (7–50 IU/L). In the peripheral smear, scattered platelet clumps and large platelets were noted, and no evidence for left shifted myeloid or nucleated red cells was found.

As this patient was having positive B‐symptoms, our initial thought was that he may suffer from a malignant disease. As prostate cancer is the most common malignant disorder in older male patients, we decided to perform a PSA screening test which came back positive with a level of 31 ng/mL (normal: <4.0 ng/mL). In the CT scan, he was found to have a significantly enlarged prostate with increase in size of the right inguinal lymph nodes (Figs. 1 and 2). In the bone scintigraphy (Tc‐99m MDP), he was found to have multifocal osseous metastases in the axial and proximal appendicular skeleton. The MRI DWIBS and STIR‐weighted studies confirmed multiple 5‐ to 35‐mm focal lesions scattered throughout the spine, pelvis, bilateral femoral shafts, scapulae, clavicle, ribs, and the sternum (Figs. 3 and 4). The FDG‐PET/PET‐CT revealed at least seven active focal bone lesions in the spine and pelvis with a SUV between 2.8 and 3.2 and two active lymph nodes in the right lower quadrant and lateral pelvis with a maximum SUV of 6.3.

**Figure 1:**
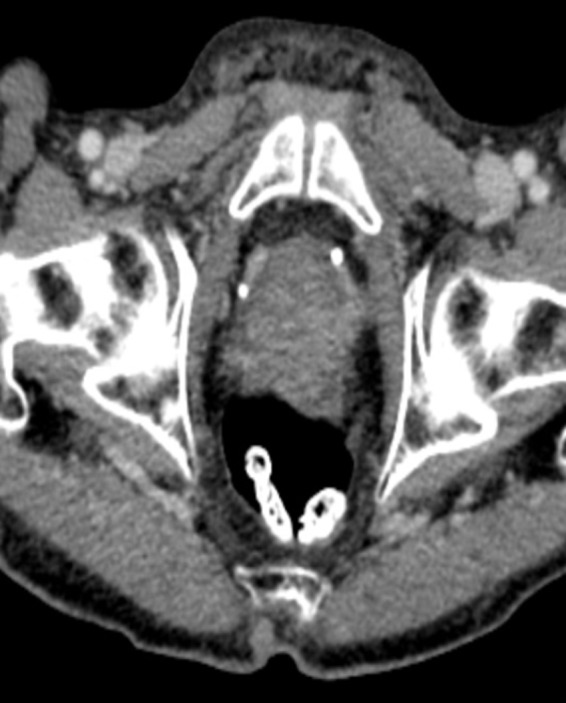
Axial postcontrast CT shows mild‐to‐moderate enlargement of the prostate gland.

**Figure 2:**
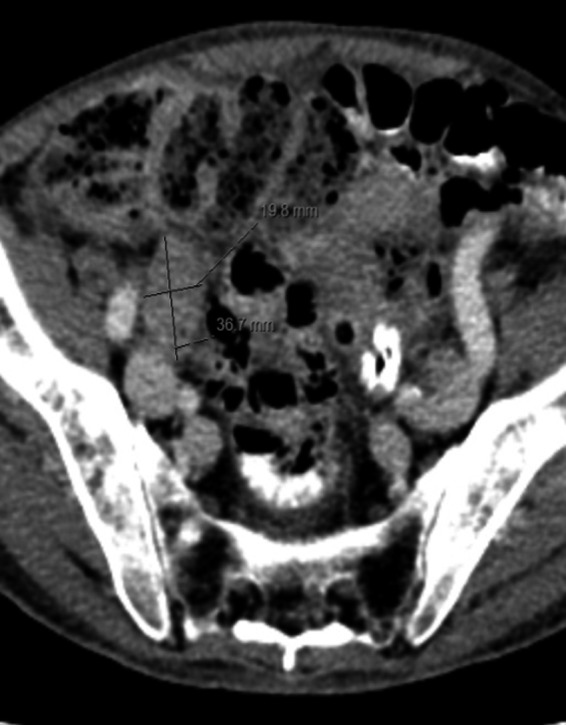
Axial postcontrast CT shows enlarged right iliac lymph node.

**Figure 3:**
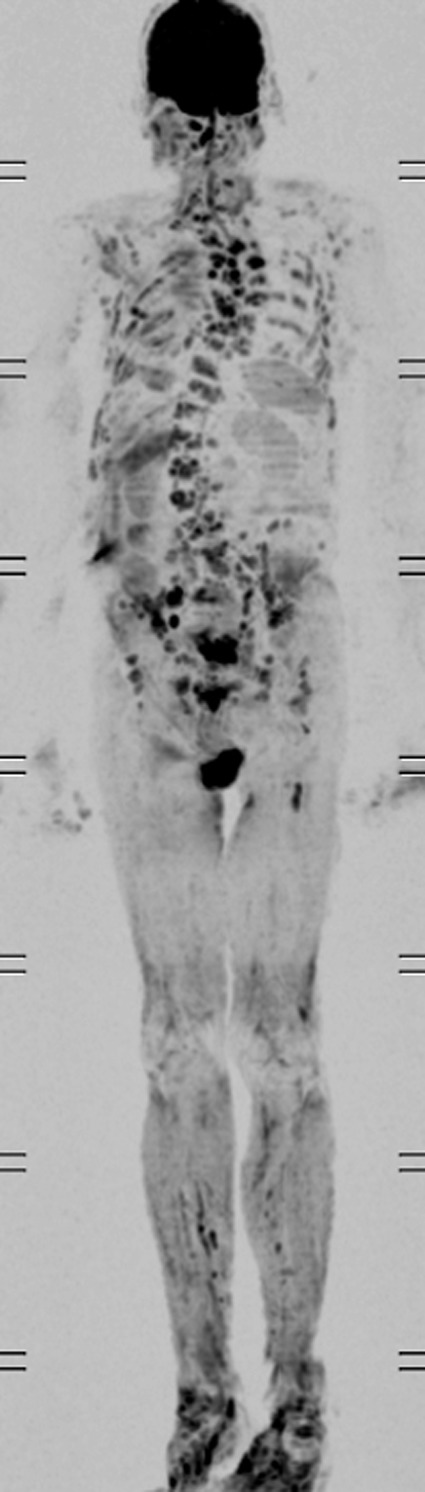
MRI DWIBS shows multiple lesions throughout the thoracic and lumbar spine. Differentiation between multiple myeloma and metastatic prostate cancer is difficult.

**Figure 4:**
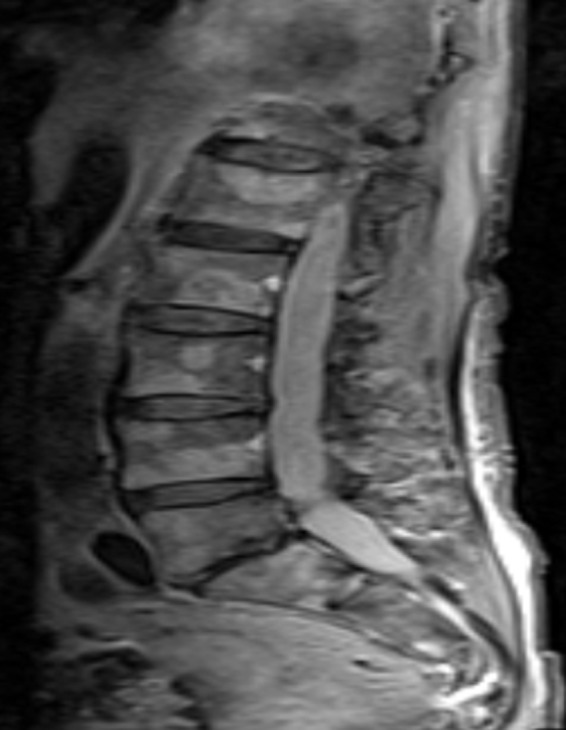
MRI STIR sagittal image of the lumbar spine shows multiple STIR hyperintense lesions. Differentiation between multiple myeloma and metastatic prostate cancer is difficult.

Interestingly, in the laboratory workup, the patient was also found to have a serum M‐protein of 2.0 g/dL (normal: 0 g/dL), Bence‐Jones proteinuria of 20 mg/24 h (normal: 0 mg/24 h), and positive immunofixation for IgG kappa M‐protein in serum and urine plus free kappa light chains (KLC) in urine, IgG‐level of 2480 mg/dL (normal: 700–1600 mg/dL) and KLC of 8.68 mg/dL (normal: 0.33–1.94 mg/dL). These findings were indicating that this patient was having not only a metastatic prostate cancer, but also concomitant diagnosis of a monoclonal plasma cell disorder. That is why we performed a bone marrow aspiration and biopsy which revealed 10–15% kappa‐expressing plasma cells (CD138+) on core biopsy with also involvement of metastatic carcinoma cells comprising approximately 10% of the core biopsy (Fig. 5; panels A, B, C, D). The area of carcinoma cells displayed dense fibrosis with clusters of large epithelial cells with open chromatin, prominent nucleoli, and high mitotic rate. The cancer cells were positive for pancytokeratin (Fig. 5; panel E), weak positive for CD138 (panel C), and focally positive for P504S (Fig. 5; panel F), indicating the diagnosis of metastatic prostate cancer and making a prostate biopsy redundant according to the urologists.

**Figure 5:**
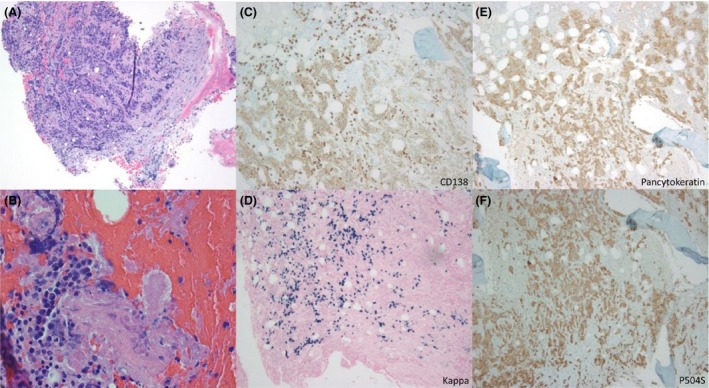
Bone marrow biopsy revealing 10–15% kappa‐expressing plasma cells (CD138+) on core biopsy (panels A, B, C, D) with involvement of prostatic carcinoma cells comprising approximately 10% of the core biopsy (panels A, B, E, F).

We came to conclusion that the patient had diagnosis of de novo metastatic (lymph nodes, bones, bone marrow) hormonal‐sensitive prostate cancer. According to the International Myeloma Working Group (IMWG) criteria, he was concomitantly also revealing at least the diagnosis of smoldering myeloma as he was revealing monoclonal paraprotein in the serum and urine with >10% myeloma cells in the bone marrow 1. Smoldering myeloma usually only requires a watch‐and‐wait strategy 1. Treatment of smoldering myeloma is recommended if patients reveal a higher probability of transformation to multiple myeloma, which is defined by the new IWMG criteria: ≥60% clonal plasma cell in the bone marrow, ≥100 of involved serum free light chains, or ≥1 MRI‐defined focal lesion ≥5 mm 1. If a patient has end‐organ damage which is defined by the CRAB criteria (hyper*C*alcemia, *R*enal insufficiency, *A*nemia, *B*one lesions), the diagnosis of multiple myeloma is made, and specific antimyeloma treatment is required according to the IMWG guidelines 1. In this case, the patient has no hypercalcemia, no renal insufficiency, and no anemia. However, he had clear evidence for MRI‐defined bone lesions which is clear CRAB criteria for definition of multiple myeloma 1. Very frequently, bone lesions are the only criteria to differentiate between smoldering and multiple myeloma.

In this case, we would like to raise attention to the possibility of concomitant presence of metastatic prostate cancer and plasma cell disorder in older male patients. The exact differentiation between multiple myeloma and metastatic prostate cancer is challenging as bone lesions/metastases are typically seen in both tumor entities 2, 3, 4, 5, 6. Profound knowledge and experience with both tumor entities are required in order to exactly define and characterize both malignancies. We highly recommend to have an intense discussion with the radiologists to describe the bone lesions in order to clearly define the source of the bone lesions. In case of doubt, we recommend to perform a fine needle aspiration (FNA) in order to distinguish between myeloma‐ and prostate cancer‐related bone metastases/lesions.

Several cases have been reported indicating the difficulties related to concomitant diagnosis of metastatic prostate cancer and multiple myeloma in older male patients 2, 3, 4, 5, 6. Multiple myeloma is typically associated with osteolytic lesions while bone metastases related to prostate cancer is typically revealing osteosclerotic lesions (Figs. 6 and 7) 1, 7, 8, 9. The only plasma cell disorder which is associated with osteosclerotic bone lesions is POEMS‐(*P*olyneuropathy, *O*rganomegaly, *E*ndocrinopathy, *M*onoclonal gammopathy, *S*kin changes) syndrome 10. In our case, the patient had no other clinical evidence for POEMS‐syndrome, except the monoclonal gammopathy.

**Figure 6:**
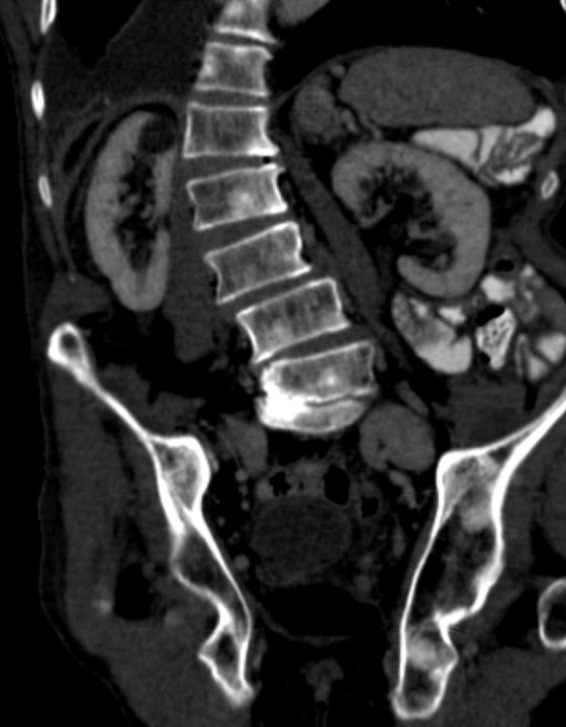
Coronal CT‐images show scattered lesions in the lumbar spine which are sclerotic in appearance. Sclerotic lesions are not typically seen in myeloma. Metastatic lesions from prostate cancer in men are in the differential diagnosis.

**Figure 7:**
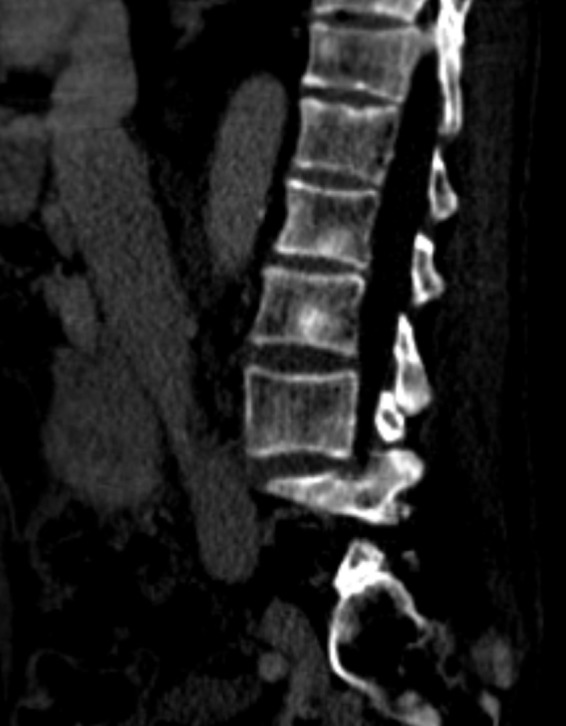
Sagittal CT‐images show scattered lesions in the lumbar spine which are sclerotic in appearance. Sclerotic lesions are not typically seen in myeloma. Metastatic lesions from prostate cancer in men are in the differential diagnosis.

He was also having no hypercalcemia which is typically seen in myeloma patients, as bone destruction and lysis are leading to hypercalcemia 1. Moreover, as seen in this case, loco‐regional lymph node metastases are typically found in advanced metastatic prostate cancer and not in multiple myeloma (Fig. 2). Lymph node infiltration in myeloma which is classified as extramedullary disease is not commonly seen 11. After a discussion with our radiologists, we came to conclusion that the focal bone lesions were characteristic of bone and lymph node metastasis due to prostate cancer.

The patient was diagnosed with de novo metastatic, hormonal‐sensitive prostate cancer with bone marrow infiltration and IgG kappa smoldering myeloma. The patient was referred to our oncologists for further palliative treatment of the prostate cancer. We decided to do a watch‐and‐wait strategy regarding the smoldering myeloma. The myeloma markers in serum and urine remained stable over the following months which supported our hypothesis that this patient had smoldering myeloma. Despite effective systemic androgen deprivation therapy (bicalutamide, leuprolide, enzalutamide, abiraterone) with significant decrease in the testosterone level (<10 ng/dL) and treatment with radium‐223, the patient deceased after 12 months due to rapid progression of his metastatic prostate cancer with significant increase in the PSA levels from 31 ng/mL to 324 ng/mL and size of bone and lymph node metastases.

## Authorship

PM was involved in patient care and writing the manuscript. DA has reviewed and arranged the pathology images. MK has reviewed and arranged the radiology images. ST was responsible for the concept of this case report. ST has analyzed, interpreted the test results, and critically reviewed the manuscript.

## Conflict of Interest

None declared.
